# MALDI-TOF Mass Spectrometry-Based Optochin Susceptibility Testing for Differentiation of *Streptococcus pneumoniae* from other *Streptococcus mitis* Group Streptococci †

**DOI:** 10.3390/microorganisms9102010

**Published:** 2021-09-23

**Authors:** Ilka D. Nix, Evgeny A. Idelevich, Andreas Schlattmann, Katrin Sparbier, Markus Kostrzewa, Karsten Becker

**Affiliations:** 1 Institute of Medical Microbiology, University Hospital Münster, 48149 Münster, Germany; ilka.nix@bruker.com (I.D.N.); evgeny.idelevich@med.uni-greifswald.de (E.A.I.); a.schlattmann@gmx.net (A.S.); 2 Bruker Daltonics GmbH & Co. KG, 28359 Bremen, Germany; katrin.sparbier@bruker.com (K.S.); markus.kostrzewa@bruker.com (M.K.); 3 Friedrich Loeffler-Institute of Medical Microbiology, University Medicine Greifswald, 17475 Greifswald, Germany

**Keywords:** *Streptococcus pneumoniae*, MALDI-TOF mass spectrometry, optochin susceptibility testing, rapid test, DOT-MGA

## Abstract

Discrimination of *Streptococcus pneumoniae* from other *Streptococcus mitis* group (SMG) species is still challenging but very important due to their different pathogenic potential. In this study, we aimed to develop a matrix-assisted laser desorption/ionization time-of-flight mass spectrometry (MALDI-TOF MS)-based optochin susceptibility test with an objective read-out. Optimal test performance was established and evaluated by testing consecutively collected respiratory isolates. Optochin in different concentrations as a potential breakpoint concentration was added to a standardized inoculum. Droplets of 6 µL with optochin and, as growth control, without optochin were spotted onto a MALDI target. Targets were incubated in a humidity chamber, followed by medium removal and on-target protein extraction with formic acid before adding matrix with an internal standard. Spectra were acquired, and results were interpreted as *S. pneumoniae* in the case of optochin susceptibility (no growth), or as non-*S. pneumoniae* in the case of optochin non-susceptibility (growth). Highest test accuracy was achieved after 20 h incubation time (95.7%). Rapid testing after 12 h incubation time (optochin breakpoint 2 µg/mL; correct classification 100%, validity 62.5%) requires improvement by optimization of assay conditions. The feasibility of the MALDI-TOF MS-based optochin susceptibility test was demonstrated in this proof-of-principle study; however, confirmation and further improvements are warranted.

## 1. Introduction

The genus *Streptococcus* comprises more than 100 different species and it is to be expected that further species will be discovered [1]. Differentiation of *Streptococcus pneumoniae* from other *Streptococcus mitis* group (SMG) species in clinical diagnostics is still challenging, but of major relevance due to their different pathogenic potential [1]. Various experimental approaches for discrimination of *S. pneumoniae*, e.g., bile solubility, optochin susceptibility, biochemical or molecular methods, are applied in clinical diagnostics, with their respective advantages and limitations [1]. Even the matrix-assisted laser desorption/ionization time-of-flight mass spectrometry (MALDI-TOF MS) as an accurate and rapid method has limitations in differentiating *S. pneumoniae* from other SMG streptococci [1,2,3,4,5,6,7,8]. As a MALDI-TOF MS instrument is now available in many diagnostic laboratories [9,10,11,12,13], it is useful to develop further assays on the same platform, besides standard species identification. Since most *S. pneumoniae* isolates are optochin susceptible [1], optochin susceptibility testing by disk diffusion is recommended by WHO for discrimination of *S. pneumoniae* [14]. However, no consensus guidelines exist for standardized performing and interpreting of the assay [15]. In this study, we aimed to investigate whether the differentiation of pneumococci from other SMG members is possible using the recently described MALDI-TOF MS-based direct-on-target microdroplet growth assay (DOT-MGA) [16]. We thereby sought to determine optimal test conditions and workflow, followed by confirmation of these assay conditions, testing consecutively collected clinical isolates.

## 2. Materials and Methods

### 2.1. Bacterial Strains

Five reference strains (*S. pneumoniae* ATCC 20566, *S. pneumoniae* ATCC 49619, *S. mitis* DSM 12643, *S. pseudopneumoniae* DSM 18670, *S. oralis* DSM 20627), as well as eight consecutively collected blood culture isolates from clinical diagnostics of the Institute of Medical Microbiology (University Hospital Münster, Münster, Germany) [17] comprising four pneumococci and four SMG streptococci (Table S2), were used to establish optimal test conditions and for determination of optochin breakpoint concentration (development set). In an additional confirmation study, 24 consecutive respiratory isolates from clinical diagnostics of the Institute of Medical Microbiology of the University Hospital Münster, including 12 pneumococci and 12 other SMG streptococci (Table S3), were used to evaluate test performance (test set). Only one isolate per patient was included. Cultivation of isolates was carried out on Columbia Blood Agar at 35 ± 1 °C in 5% CO_2_.

### 2.2. Species Identification by Molecular and Biochemical Method

Species identification was performed by standard molecular methods. *sodA* gene sequencing was carried out for confirmation of streptococcal isolates. *rpoB* gene sequencing was performed for differentiation between *S. pneumoniae* and *S. pseudopneumoniae*. Autolysin (*lytA*) and pneumolysin (*ply*) were detected using endpoint PCR. PCR amplification protocols and primer sequences were used as previously described [17]. NCBI’s Basic Local Alignment Search Tool [18] was used for analysis of sequences. Additionally, biochemical identification of all isolates was performed by the Vitek 2 instrument using GP ID cards (#21342, bioMérieux, Marcy l’Etoile, France) according to the manufacturer’s instructions.

### 2.3. Bile Solubility Testing

A classical bile solubility tube test and bile solubility plate test were performed as previously described [1]. Briefly, for the bile solubility tube test, 0.5 mL of a McFarland 0.5–1.0 suspension in 0.9% NaCl was mixed with 0.5 mL of 10% sodium deoxycholate (Sigma Aldrich, Darmstadt, Germany) in a small tube. As a control, 0.5 mL of bacterial suspension was mixed with 0.5 mL 0.9% NaCl. Tubes were incubated at 35 ± 1 °C. A positive result, i.e., clearing of the bacterial suspension within 3 h, defined the identification as *S. pneumoniae*. Because clearing can start as early as 15 min after inoculation, results were visually read after 30 min and after 3 h. Due to the difficulties in reading bile solubility results, tests were additionally performed with a McFarland suspension of 4.0. For the bile solubility plate test, a single colony on an agar plate was overlaid with one drop of 10% sodium deoxycholate. The plate was incubated at 35 ± 1 °C for 15 to 30 min in ambient air. Colonies of *S. pneumoniae* disappeared or showed a flattened morphology; the appearance of non-*S. pneumoniae* colonies did not change.

### 2.4. Optochin Susceptibility Testing by Disk Diffusion

Additionally, optochin susceptibility testing by disk diffusion was performed as previously described [1]. Briefly, McFarland 0.5 suspension in 0.9% NaCl was streaked on a blood agar plate. Subsequently, 9 mm disks containing 23 µg of optochin (Optochin Test OPTO-F, #55912, bioMérieux, Marcy l’Etoile, France) were placed onto the blood agar plates and incubated at 35 ± 1 °C overnight in 5% CO_2_ and in ambient air environments. According to the manufacturer’s instructions (bioMérieux, Marcy l’Etoile, France), inhibition zones of ≥15 mm defined susceptibility, confirming *S. pneumoniae* identification.

### 2.5. Determination of Minimum Inhibitory Concentration (MIC)

Because of a lack of a valid optochin breakpoint concentration to distinguish between optochin-susceptible and non-susceptible isolates, resulting in *S. pneumoniae* or non-*S. pneumoniae* discrimination, minimum inhibitory concentrations (MICs) of optochin were determined for test development. MIC determination was performed by broth microdilution according to the current CLSI guidelines [19,20]. In brief, overnight cultures on blood agar (35 ± 1 °C, 5% CO_2_) were used to prepare a standardized inoculum of McFarland 0.5 turbidity in 0.9% NaCl. Standardized bacterial suspension was diluted to 10^−2^ in cation-adjusted Mueller–Hinton broth with lysed horse blood (CAMHB with LHB (CP112-10), Thermo Scientific™/Remel Inc., San Diego, CA, USA). Optochin (ethylhydrocupreine hydrochloride, Sigma Aldrich, Darmstadt, Germany) concentrations in the range of 0.125–256 µg/mL were tested in double dilution steps. A final inoculum of approximately 5 × 10^5^ cfu/mL was generated using 50 µL of diluted bacterial suspension mixed with 50 µL of optochin solution in a 96-well microtiter plate. Inoculum quantity was confirmed by vital cell counting of serial dilutions onto blood agar in triplicates after overnight incubation at 35 ± 1 °C in 5% CO_2_. After 20–24 h incubation at 35 ± 1 °C in ambient air, results of broth microdilution were visually read. All tests were performed in triplicate and the median MIC was determined.

### 2.6. MALDI-TOF MS-Based Optochin Susceptibility Test

#### 2.6.1. Investigation of Optimal Test Conditions

Investigation of optimal test conditions for MALDI-TOF MS-based optochin susceptibility test was carried out using five reference strains and eight consecutively collected clinical blood culture isolates (*n* = 13, development set). DOT-MGA was performed as previously described [16,21,22]. In brief, a bacterial suspension with turbidity of 0.5 McFarland standard was prepared from an overnight culture on blood agar. For growth control, this suspension was diluted to 10^−2^ in CAMHB with LHB and added to the same volume of CAMHB with LHB. Additionally, the diluted bacterial suspension was added to the same volume of CAMHB with LHB containing optochin in final concentrations of 2, 4 or 8 µg/mL, as possible breakpoint concentrations dividing between optochin susceptible and non-susceptible isolates. The final inoculum of approximately 5 × 10^5^ cfu/mL was confirmed by vital cell counting, as described above. Droplets of 6 µL were transferred from each well of the microtiter plate onto a disposable MALDI target (MBT Biotarget 96, Bruker Daltonics GmbH & Co. KG, Bremen, Germany) in duplicate (double spotting). For each time point, a separate target was prepared. The targets were incubated in a plastic transport box (Bruker Daltonics GmbH & Co. KG, Bremen, Germany), where 4 mL of water were added to generate a humid atmosphere and to avoid evaporation of microdroplets. The boxes were incubated for 8 and 24 h at 35 ± 1 °C. An additional target was incubated for 24 h in an incubation chamber device with individual temperature control, which results in standardized incubation conditions and minimal risk of evaporation (MBT FAST shuttle prototype (Bruker Daltonics GmbH & Co. KG, Bremen, Germany)). After incubation, the liquid medium was removed to avoid interference with broth ingredients during MALDI-TOF MS measurement. For targets that were incubated in the plastic transport box, medium was removed using novel absorptive pads (Bruker Daltonics GmbH & Co. KG, Bremen, Germany) while touching the droplets carefully from the top. For targets that were incubated in the MBT FAST shuttle prototype, a stamp device filled with an absorptive pad and fit to the incubation chamber device was used to remove the medium (MBT FAST stamp prototype (Bruker Daltonics GmbH & Co. KG, Bremen, Germany)). The dried spots on the target were overlaid with 1 μL 70% formic acid. Subsequently, the dried spots were overlaid with 1 μL matrix spiked with an internal standard (MBT FAST matrix (Bruker Daltonics GmbH & Co. KG, Bremen, Germany)) dissolved in the standard solvent (Solution OS, LCH CHIMIE, Les Aires, France) according to the manufacturer’s instructions.

#### 2.6.2. Confirmation of Test Conditions and Evaluation of Assay Performance

Established test conditions were confirmed in an additional confirmation study using 12 pneumococci and 12 other SMG clinical isolates (*n* = 24, test set). A MALDI-TOF MS-based optochin susceptibility test was performed exactly as described above. Optochin breakpoint concentrations of 2, 4 and 8 µg/mL were further tested in this study part, because previous results could not clearly demonstrate an optimal concentration that distinguishes between optochin-susceptible and non-susceptible isolates. Incubation times of 8, 10, 12 and 20 h in ambient air were tested, while targets were placed in a plastic transport box filled with 4 mL water at the bottom. An additional target was incubated for 20 h in the MBT FAST shuttle prototype (Bruker Daltonics GmbH & Co. KG, Bremen, Germany). For testing *S. pseudopneumoniae* isolates, targets were additionally incubated with 5% CO_2_ in plastic transport boxes for 8, 10, 12 and 20 h at 35 ± 1 °C. After incubation, the medium was removed from all targets using the MBT FAST stamp with absorptive pads. Subsequent target preparation was carried out as described above.

#### 2.6.3. Spectrum Acquisition, Determination of Specific Cut-Off Values and Data Evaluation

MALDI-TOF MS measurement was performed using the MALDI Biotyper smart instrument (Bruker Daltonics GmbH & Co. KG, Bremen, Germany) and the flexControl software (Version 3.4, Bruker Daltonics GmbH & Co. KG, Bremen, Germany). Spectra were acquired with optimized instrument settings for the MBT FAST assay and a modified AutoXecute method (*MBT-FAST.axe*) was used. Each spot was measured twice. Acquired spectra were analyzed using a novel prototype software (MBT FAST prototype software), which compared the acquired spectra on spots with antibiotics to the spectra of the corresponding growth control; for test development, various evaluation criteria were tested to categorize the acquired spectra as representing optochin susceptibility (no growth means identification as *S. pneumoniae*) or optochin non-susceptibility (growth means identification as non-*S. pneumoniae*). The evaluation criteria that performed best for the isolates of the development set were applied for evaluation of the test set isolates in the confirmation part of the study. Tests were considered valid if the growth control was successfully detected. Molecular characterization was used as a reference identification method, and rates of false-positive and false-negative classifications were calculated for valid tests. False-negative rate was the proportion of isolates tested as optochin non-susceptible (non-*S. pneumoniae*) by MALDI-TOF MS DOT-MGA but identified as *S. pneumoniae* by the molecular reference method. False-positive rate was the proportion of isolates tested as optochin susceptible (*S. pneumoniae*) by MALDI-TOF MS DOT-MGA but identified as non-*S. pneumoniae* by the reference method.

## 3. Results

### 3.1. Molecular and Biochemical Identification Methods

The results of molecular and biochemical identification for development set isolates are shown in Table S1. The results of the classical bile solubility and optochin susceptibility testing by disk diffusion corresponded to the molecular identification results, but with limitations in correct identification of *S. pseudopneumoniae* (Table S1). Twenty-four clinical isolates of the test set were confirmed as 12 *S. pneumoniae* and 12 SMG isolates by molecular and biochemical identification, as well as classical bile solubility and optochin susceptibility by disk diffusion, but there were also difficulties in correct identification of *S. pseudopneumoniae* (Table S2).

### 3.2. Minimum Inhibitory Concentration and Optochin Breakpoint Determination

**Figure 1:**
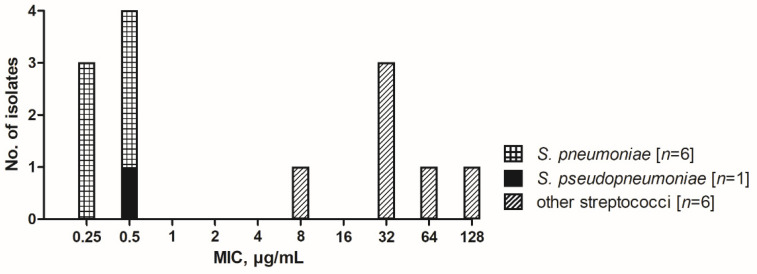
Distribution of MICs within the development set isolates to determine an optimal optochin breakpoint concentration for following test development.

MICs of optochin for the development set isolates are presented in Table S3. There was a clear delineation of optochin MICs for *S. pneumoniae* and *S. pseudopneumoniae* (when the latter was tested in ambient air), on the one hand, and MICs of other SMG species, on the other hand (Figure 1). For following test development, 2, 4 and 8 µg/mL optochin were chosen as potential breakpoint concentrations to distinguish between pneumococci and non-pneumococci.

### 3.3. Investigation of Optimal Test Conditions

In total, 13 streptococci isolates (development set) were tested with three different optochin concentrations (2, 4 and 8 µg/mL). Software configurations based on the tested strain set resulting in best overall results were used for the final evaluation of assay performance. All tested optochin concentrations showed comparable results and, compared to molecular methods used as a reference, all tested isolates were classified correctly after 24 h incubation time in the MBT FAST shuttle (Table 1). Test validity was 100% after 24 h incubation time, independent of the incubation device (Table 1). Correct classification was slightly better when targets were incubated in the MBT FAST shuttle compared to those incubated in the plastic target box. Short-time incubation showed no sufficient validity; only 23.1% of tested isolates showed sufficient growth after 8 h incubation time (Table 1).

### 3.4. Confirmation of Test Conditions and Evaluation of Assay Performance

Experiments on test development showed no clear optochin breakpoint concentration, so that 2, 4 and 8 µg/mL optochin concentrations were further tested in the confirmation study part as candidate breakpoints. Overall, 24 clinical streptococci isolates were tested and with 20 h incubation time, test validity was—independent of incubation device—95.8% (Table 2). Correct classification of *S. pneumoniae* and non-*S. pneumoniae* isolates was at least 91.3% (2 µg/mL optochin and incubation in MBT-FAST shuttle) and 100% for 2 µg/mL optochin breakpoint concentration when incubated in the plastic target transport box (Table 2).

Short incubation times showed sufficient growth (test validity) in 62.5%, 37.5% and 20.8% of isolates after 12, 10 and 8 h, respectively (Table 3). The best result for differentiation of pneumococci at earlier time points was achieved when applying 2 µg/mL optochin as the breakpoint concentration. In this setting, correct classification was 100% (validity 62.5%) after 12 h (Table 3). Applying 4 and 8 µg/mL optochin, correct classification was achieved in 93.3% and 86.7% after 12 h’ incubation, respectively (Table 3).

In accordance with the guidelines for susceptibility testing of streptococci [19,20], optochin susceptibility testing was carried out at 35 ± 1 °C in ambient air. However, for differentiation of *S. pneumoniae* and *S. pseudopneumoniae*, MALDI-TOF MS-based optochin susceptibility testing was additionally performed with incubation of *S. pseudopneumoniae* isolates at 35 ± 1 °C in 5% CO_2_. *S. pseudopneumoniae* were correctly classified only after 20 h incubation time and with 2 µg/mL optochin as the breakpoint concentration.

## 4. Discussion

Many clinical laboratories have implemented MALDI-TOF MS for rapid identification of microorganisms [9,10,11,12,13]. It would be advantageous and cost efficient to run additional diagnostic tests on the same MALDI-TOF MS instrument. The recently developed MALDI-TOF MS-based DOT-MGA is a rapid and universal antimicrobial susceptibility testing method, which can be easily integrated in daily diagnostic workflows [16,23].

Limitations of conventional MALDI-TOF MS identification in distinguishing *S. pneumoniae* from other SMG streptococci have been shown in several studies [1,2,3,4,5,6,7,8]. That is why we developed a MALDI-TOF MS-based optochin susceptibility test as a complementary confirmation method for discrimination between pneumococci and non-pneumococci.

For test development, MIC results of the development set isolates were used to determine an optochin breakpoint concentration for distinguishing between susceptible and resistant isolates, as no valid breakpoint is available for optochin broth microdilution testing [20,24]. MIC distribution of the development set isolates showed a clear distinction between optochin-susceptible *S. pneumoniae* (MIC range 0.25–0.5 µg/mL) and optochin-resistant non-*S. pneumoniae* (MIC range 8–128 µg/mL) (Table S3). Only one *S. mitis* isolate showed an MIC of 8 µg/mL, whereas optochin MICs for all other non-*S. pneumoniae* isolates were between 32 and 128 µg/mL (Figure 1 and Table S3). The reference strain of *S. pseudopneumoniae* showed optochin susceptibility (MIC 0.5 µg/mL, Figure 1 and Table S3), when incubated in ambient air [25]. As candidate breakpoint concentrations for testing in the MALDI-TOF MS-based DOT-MGA, 2, 4 and 8 µg/mL optochin were chosen.

Results of the investigation of optimal test conditions showed that an 8 h incubation time was too short for sufficient growth of SMG isolates (Table 1). In contrast, a 24 h incubation time showed satisfactory results that were independent of incubation device (plastic box or MBT FAST shuttle). Minimal differences between incubation devices might be due to variability because of the relatively low number of isolates in this proof-of-concept study. This study phase showed the principal feasibility of the MALDI-TOF MS-based optochin testing but revealed limitations of short incubation times due to the slow growth of SMG species. There was no remarkable difference in results achieved with different optochin breakpoint concentrations; however, testing with optochin concentration of 8 µg/mL yielded more false-negative results after 24 h incubation in plastic box (7.7%). The reason for this could be the worse quality of the manual supernatant removal in comparison to the supernatant removal with the MBT FAST stamp prototype. Therefore, in the following confirmation study phase, culture supernatant was removed using the MBT FAST stamp prototype independent of incubation device (plastic box or MBT FAST shuttle prototype). Moreover, 2, 4 and 8 µg/mL optochin were further tested in parallel, because no clear decision could be made after the investigation of optimal test conditions.

For the confirmation study, incubation times of 8, 10 and 12 h were tested, as well as 20 h, which is the recommended time for standard broth microdilution susceptibility testing of streptococci [19,20,24,26]. Results with shorter incubation times confirmed difficulties in rapid differentiation of streptococci (Table 3). Nevertheless, a 12 h incubation time showed promising results (62.5% validity and 100% correct classification), although test conditions should be further optimized in future studies including larger strain collections. Acceleration of growth detection could be achieved, e.g., by improved media composition, inoculum size, and software algorithms. Due to usual working hours in clinical laboratories, test periods of more than 8 h could be difficult to integrate into the daily workflow. Results achieved after 20 h incubation in the confirmation study part were very promising (Table 2), as 95.8% validity indicated that this time is enough for sufficient growth.

The false-positive rate was identical for different optochin concentrations and incubation devices when the incubation time was 20 h (Table 2). For shorter incubation times, the false-positive rate increased with increasing optochin concentrations, indicating that higher concentrations of optochin inhibited microbial growth (Table 2). Therefore, 2 µg/mL optochin was the optimal breakpoint concentration, when applying short incubation. This is in accordance with the MICs distribution of test set isolates (Table S3). Most *S. pneumoniae* and *S. pseudopneumoniae* isolates (for the latter, if MIC determination performed in ambient air) showed MICs < 2 µg/mL.

*S. pseudopneumoniae* has been reported to be insoluble in bile, as well as resistant or intermediate susceptible to optochin when incubated in 5% CO_2_, but susceptible to optochin when incubated in ambient air [25]. According to the current guidelines [19], susceptibility testing of streptococci should be performed in ambient air. In our study, MICs were determined under ambient air conditions during the investigation of optimal test conditions. During the evaluation of assay performance, MALDI-TOF MS-based DOT-MGA setup for *S. pseudopneumoniae* isolates of the test set (*n* = 2) was additionally incubated in 5% CO_2_ (data not shown). The growth of *S. pseudopneumoniae* was already sufficient after 8 h of incubation time in 5% CO_2_ (100% validity), which was not the case when incubated in ambient air (Table 3). Correct classification of *S. pseudopneumoniae* isolates was achieved after 20 h incubation in 5% CO_2_ with 2 µg/mL optochin as the breakpoint concentration, taking into account that *S. pseudopneumoniae* is known to be resistant to optochin when tested in 5% CO_2_ by the standard method [25].

While molecular identification was used as the reference method in this study, other standard differentiation methods were also evaluated. The results of the classical bile solubility test using 0.5 McFarland suspension were partly difficult to read (Tables S1 and S2). The results could be read better when the test was repeated with a 4.0 McFarland suspension; however, there still were interpretation difficulties due to subjective judgment. The bile insolubility of *S. pseudopneumoniae* [25] could not be confirmed due to ambiguous results (Tables S1 and S2). Optochin susceptibility testing by disk diffusion performed in parallel in ambient air and in 5% CO_2_ did not clearly show the previously reported effect of incubation in 5% CO_2_ [25,27,28] (Table S2). Biochemical identification by the Vitek 2 instrument was not able to distinguish between *S. pneumoniae* and *S. pseudopneumoniae* (Tables S1 and S2).

Different methods for discrimination of pneumococci possess various advantages and limitations. Molecular identification can be considered as a reference method, but is complex, time consuming and expensive [29]. Biochemical testing has limitations in pneumococci differentiation [30,31], as does MALDI-TOF MS [3,4,5]. Bile solubility is a rapid method for discrimination of pneumococci, which is however limited by subjective visual interpretation and thereby, frequent inconclusive results [32,33]. Recently, Idelevich et al. suggested a novel MALDI-TOF MS-based bile solubility test with an objective read-out [17]. Although the bile solubility test is more specific than the optochin test for discrimination of *S. pneumoniae* [27,28], classical optochin susceptibility assay by disk diffusion is largely used in diagnostic laboratories, probably because of simple visual interpretation [34]. Nevertheless, optochin susceptibility performed as a classical disk diffusion assay also has limitations, and requires overnight incubation [1]. Notably, Burckhardt et al. demonstrated that automated reading of disk diffusion optochin susceptibility tests is feasible after only 12 h of incubation [15]. However, manual reading after 12 h incubation can hardly be integrated into the laboratory workflow, as most European laboratories are not manned 24/7 [13].

## 5. Conclusions

This study showed that optochin susceptibility testing by an alternative MALDI-TOF MS-based approach is feasible within 20 h’ incubation time. A short time incubation was, however, not sufficient due to the slow growth of streptococci. It remains to be investigated if the improvement of assay conditions can considerably reduce the test duration. This suggested assay could be used as a confirmation assay for standard methods, which still have limitations and need confirmation, due to the different pathogenic potential of *S. pneumoniae* and other SMG streptococci.

## Figures and Tables

**Table 1:** Performance of MALDI-TOF MS-based DOT-MGA for establishing optimal test conditions for optochin susceptibility testing using evaluation with MBT FAST prototype ^a^.

Incubation Type	Plastic Target Box						MBT FAST Shuttle (Prototype)		
Incubation Time	8 h			24 h			24 h		
Optochin Concentration	2 µg/mL	4 µg/mL	8 µg/mL	2 µg/mL	4 µg/mL	8 µg/mL	2 µg/mL	4 µg/mL	8 µg/mL
Validity [%]	23.1	23.1	23.1	100	100	100	100	100	100
Correct classification ^b^ [%]	66.7	66.7	66.7	92.3	92.3	84.6	100	100	100
False negative ^b^ [%]	0.0	0.0	0.0	0.0	0.0	7.7	0.0	0.0	0.0
False positive ^b^ [%]	33.3	33.3	33.3	7.7	7.7	7.7	0.0	0.0	0.0

^a^ development set isolates, *n* = 13; ^b^ Calculated for valid tests.

**Table 2:** Accuracy of the MALDI-TOF MS-based optochin susceptibility testing after 20 h incubation time ^a^.

Incubation Device	Plastic Target Box			MBT FAST Shuttle (Prototype)		
Incubation Time	20 h			20 h		
Optochin Concentration	2 µg/mL	4 µg/mL	8 µg/mL	2 µg/mL	4 µg/mL	8 µg/mL
Validity [%]	95.8	95.8	95.8	95.8	95.8	95.8
Correct classification ^b^ [%]	100	95.7	95.7	91.3	95.7	95.7
False negative ^b^ [%]	0.0	0.0	0.0	4.3	0.0	0.0
False positive ^b^ [%]	0.0	4.3	4.3	4.3	4.3	4.3

^a^ test set isolates, *n* = 24; ^b^ Calculated for valid tests.

**Table 3:** Accuracy of the MALDI-TOF MS-based optochin susceptibility testing after short incubation ^a^.

Incubation Type	Plastic Target Box								
Incubation Time	8 h			10 h			12 h		
Optochin Concentration	2 µg/mL	4 µg/mL	8 µg/mL	2 µg/mL	4 µg/mL	8 µg/mL	2 µg/mL	4 µg/mL	8 µg/mL
Validity [%]	20.8	20.8	20.8	37.5	37.5	37.5	62.5	62.5	62.5
Correct classification ^b^ [%]	40.0	40.0	20.0	100	88.9	66.7	100	93.3	86.7
False negative ^b^ [%]	0.0	0.0	0.0	0.0	0.0	0.0	0.0	0.0	0.0
False positive ^b^ [%]	60.0	60.0	80.0	0.0	11.1	33.3	0.0	6.7	13.3

^a^ test set isolates, *n* = 24; ^b^ Calculated for valid tests.

## Data Availability

Data are contained within the article or Supplementary Material.

## Supplementary Materials

The following is available online at https://www.mdpi.com/article/10.3390/microorganisms9102010/s1, Table S1: Results of molecular/biochemical species identification and bile solubility/optochin disk diffusion for the development set of isolates (*n* = 13). Table S2: Results of molecular/biochemical species identification and bile solubility/optochin disk diffusion for the test set of isolates (*n* = 24). Table S3: Results of optochin minimum inhibitory concentration (MIC) for the development set (*n* = 13) and the test set (*n* = 24) isolates.
