# Supplementary material for: MALDI-TOF Mass Spectrometry-Based Optochin Susceptibility Testing for Differentiation of Streptococcus pneumoniae from other Streptococcus mitis Group Streptococci
Source: Microorganisms. 2021 Sep 23;9(10):2010. doi: 10.3390/microorganisms9102010 (PMC8537049; doi:10.3390/microorganisms9102010)
Supplement: Supplementary file 1 [file microorganisms-09-02010-s001.zip › microorganisms-1386107-supplementary.pdf]

# MALDI-TOF mass spectrometry-based optochin susceptibility testing for differentiation of *Streptococcus pneumoniae* from other *Streptococcus mitis* group streptococci

Ilka D. Nix, Evgeny A. Idelevich, Andreas Schlattmann, Katrin Sparbier,
Markus Kostrzewa, Karsten Becker

## Supplementary Materials

**Table S1**.
Results of molecular/biochemical species identification and bile solubility/optochin disk diffusion for the development set of isolates (*n*=13).
**Table S2**.
Results of molecular/biochemical species identification and bile solubility/optochin disk diffusion for the test set of isolates (*n*=24).
**Table S3.**
Results of optochin minimum inhibitory concentration (MIC) for the development set (*n*=13) and the test set (*n*=24) isolates.

**Table S1.** Results of molecular/biochemical species identification and bile solubility/optochin disk diffusion for the development set of isolates (*n*=13).

| | # | Molecular identification | | | | Biochemical identification (VITEK 2) | | | Bile solubility | | | | | Optochin disk diffusion | | | |
|---|---|---|---|---|---|---|---|---|---|---|---|---|---|---|---|---|---|
| | | | | | | | | | Tube test McF 0.5 | | Tube test McF 4.0 | | Plate test | Incubation at 35 ± 1 °C in ambient air | | Incubation at 35 ± 1 °C with 5% $CO_2$ | |
| | | ***sodA* sequencing** | ***rpoB* sequencing** | ***lytA*** | ***ply*** | **Species** | **Qualifying message (% probability)** | **Time to result [h]** | **30 min** | **3 hours** | **30 min** | **3 hours** | | **Inhibition zone [mm]** | **Result** | **Inhibition zone [mm]** | **Result** |
| **Development set** | **2P** | *S. pneumoniae/ pseudopneumoniae* | *S. pneumoniae* | + | + | *Streptococcus pneumoniae* | Excellent (97) | 5.00 | + | + | + | + | + | 28 | S | 20 | S |
| | **5P** | *S. pneumoniae/ pseudopneumoniae* | *S. pneumoniae* | + | + | *Streptococcus pneumoniae* | Very good (94) | 5.98 | + | + | + | + | + | 32 | S | 22 | S |
| | **6P** | *S. pneumoniae/ pseudopneumoniae* | *S. pneumoniae* | + | + | *Streptococcus pneumoniae* | Good (91) | 6.05 | + | + | + | + | + | 26 | S | 24 | S |
| | **8P** | *S. pneumoniae/ pseudopneumoniae* | *S. pneumoniae* | + | + | *Streptococcus pneumoniae* | Good (92) | 6.77 | + | + | + | + | + | 28 | S | 21 | S |
| | **1M** | *S. mitis* | ND | - | - | *Streptococcus mitis/oralis* | Good (91) | 7.02 | - | - | - | - | - | - | R | - | R |
| | **3M** | *S. mitis* | ND | - | - | *Streptococcus mitis/oralis* | Very good (93) | 5.80 | ~ | ~ | ~ | + | - | - | R | - | R |
| | **4M** | *S. oralis* | ND | - | - | *Streptococcus mitis/oralis* | Excellent (99) | 5.07 | - | - | - | - | - | - | R | - | R |
| | **5M** | *S. oralis* | ND | - | - | *Streptococcus mitis/oralis* | Excellent (98) | 5.07 | - | - | - | - | - | - | R | - | R |
| | *S. pneumoniae* **DSM20566** | ND | ND | ND | ND | *Streptococcus pneumoniae* | Very good (93) | 4.98 | - | - | + | + | - | 28 | S | 26 | S |
| | *S. mitis* **DSM12643** | ND | ND | ND | ND | *Streptococcus mitis/oralis* | Good (91) | 5.97 | - | - | - | - | - | - | R | - | R |
| | *S. pseudopneumoniae* **DSM18670** | ND | ND | ND | ND | Low discrimination (repeated test: unidentified) | Low discrimination (50) (repeated test: unidentified) | 7.90 (repeated test: 2.83) | - | - | - | - | ? | 32 | S | 20 | S |
| | *S. oralis* **DSM20627** | ND | ND | ND | ND | *Streptococcus mitis/oralis* | Excellent (97) | 5.97 | - | - | - | - | - | - | R | - | R |
| | *S. pneumoniae* **ATCC49619** | ND | ND | ND | ND | *Streptococcus pneumoniae* | Excellent (97) | 2.57 | + | + | + | + | + | 22 | S | 22 | S |

+, positive; -, negative; ~, almost transparent (ambiguous result); ND, not done; ?, not readable/unclear result; R, resistant; S, susceptible

**Table S2.** Results of molecular/biochemical species identification and bile solubility/optochin disk diffusion for the test set of isolates (*n*=24).

| | # | Molecular identification | | | | Biochemical identification (VITEK 2) | | | Bile solubility | | | | | Optochin disk diffusion | | | |
|---|---|---|---|---|---|---|---|---|---|---|---|---|---|---|---|---|---|
| | | | | | | | | | Tube test McF 0.5 | | Tube test McF 4.0 | | Plate test | Incubation at 35 ± 1 °C in ambient air | | Incubation at 35 ± 1 °C with 5% $CO_2$ | |
| | | ***sodA* sequencing** | ***rpoB* sequencing** | ***lytA*** | ***ply*** | **Species** | **Qualifying message (% probability)** | **Time to result [h]** | **30 min** | **3 hours** | **30 min** | **3 hours** | | **Inhibition zone [mm]** | **Result** | **Inhibition zone [mm]** | **Result** |
| **Test set** | **R2P** | *S. pneumoniae/ pseudopneumoniae* | *S. pneumoniae* | + | + | *Streptococcus pneumoniae* | Very good (93) | 5.78 | + | + | + | + | + | 24 | S | 24 | S |
| | **R3P** | *S. pneumoniae/ pseudopneumoniae* | *S. pneumoniae* | + | + | *Streptococcus pneumoniae* | Excellent (97) | 4.83 | + | + | + | + | + | 27 | S | 25 | S |
| | **R5P** | *S. pneumoniae/ pseudopneumoniae* | *S. pneumoniae* | + | + | *Streptococcus pneumoniae* | Excellent (99) | 4.78 | + | + | + | + | + | 23 | S | 18 | S |
| | **R6P** | *S. pneumoniae/ pseudopneumoniae* | *S. pneumoniae* | + | + | *Streptococcus pneumoniae* | Excellent (99) | 4.82 | + | + | ? | ~ | + | 24 | S | 14 | R (?) |
| | **R8P** | *S. pneumoniae/ pseudopneumoniae* | *S. pneumoniae* | + | + | *Streptococcus pneumoniae* | Excellent (97) | 5.03 | + | + | + | + | + | 23 | S | 23 | S |
| | **R10P** | *S. pneumoniae/ pseudopneumoniae* | *S. pneumoniae* | + | + | *Streptococcus pneumoniae* | Very good (94) | 5.03 | + | + | + | + | + | 23 | S | 21 | S |
| | **R11P** | *S. pneumoniae/ pseudopneumoniae* | *S. pneumoniae* | + | + | *Streptococcus pneumoniae* | Excellent (97) | 3.32 | ? | + | - | ~ | + | 28 | S | 22 | S |
| | **R13P** | *S. pneumoniae/ pseudopneumoniae* | *S. pneumoniae* | + | + | *Streptococcus pneumoniae* | Good (91) | 5.77 | + | + | + | + | + | 29 | S | 25 | S |
| | **R14P** | *S. pneumoniae/ pseudopneumoniae* | *S. pneumoniae* | + | + | *Streptococcus pneumoniae* | Very good (93) | 5.98 | + | + | + | + | + | 32 | S | 22 | S |
| | **R15P** | *S. pneumoniae/ pseudopneumoniae* | *S. pneumoniae* | + | + | *Streptococcus pneumoniae* | Very good (94) | 5.98 | ? | + | + | + | + | 25 | S | 25 | S |
| | **R16P** | *S. pneumoniae/ pseudopneumoniae* | *S. pneumoniae* | + | + | *Streptococcus pneumoniae* | Very good (95) | 6 | ? | + | + | + | + | 30 | S | 22 | S |
| | **R17P** | *S. pneumoniae/ pseudopneumoniae* | *S. pneumoniae* | + | + | *Streptococcus pneumoniae* | Very good (95) | 4.78 | ? | + | + | + | ? | 28 | S | 25 | S |

+, positive; -, negative; ~, almost transparent (ambiguous result); ND, not done; ?, not readable/unclear result; R, resistant; S, susceptible

**Table S2 (continuation).** Results of molecular/biochemical species identification and bile solubility/optochin disk diffusion for the test set of isolates (*n*=24).

| | # | Molecular identification | | | | Biochemical identification (VITEK 2) | | | Bile solubility | | | | | Optochin disk diffusion | | | |
|---|---|---|---|---|---|---|---|---|---|---|---|---|---|---|---|---|---|
| | | | | | | | | | Tube test McF 0.5 | | Tube test McF 4.0 | | Plate test | Incubation at 35 ± 1 °C in ambient air | | Incubation at 35 ± 1 °C with 5% $CO_2$ | |
| | | ***sodA* sequencing** | ***rpoB* sequencing** | ***lytA*** | ***ply*** | **Species** | **Qualifying message (% probability)** | **Time to result [h]** | **30 min** | **3 hours** | **30 min** | **3 hours** | | **Inhibition zone [mm]** | **Result** | **Inhibition zone [mm]** | **Result** |
| **Test set** | **R1P** | *S. pneumoniae/ pseudopneumoniae* | *S. pseudopneumoniae* | - | + | Low discrimination (repeated test: unidentified) | Low discrimination (50) (repeated test: unidentified) | 7.90 (repeated test: 3.32) | ? | ? | - | + | ? | 24 | S | 13 | R (?) |
| | **R12P** | *S. pneumoniae/ pseudopneumoniae* | *S. pseudopneumoniae* | - | + | *Streptococcus pneumoniae* | Very good (93) | 6.98 | + | + | ~ | ~ | ? | 25 | S | 22 | S |
| | **R2M** | *S. mitis* | ND | - | - | *Streptococcus mitis/oralis* | Excellent (98) | 5.78 | - | - | - | - | - | - | R | - | R |
| | **R5M** | *S. mitis* | ND | - | - | *Streptococcus mitis/oralis* | Very good (95) | 6.97 | - | - | - | - | - | - | R | - | R |
| | **R6M** | *S. mitis* | ND | - | - | *Streptococcus mitis/oralis* | Excellent (99) | 4.8 | - | - | - | - | - | - | R | - | R |
| | **R7M** | *S. mitis* | ND | - | - | *Streptococcus mitis/oralis* | Very good (94) | 5.03 | - | - | - | - | - | - | R | - | R |
| | **R9M** | *S. oralis* | ND | - | - | *Streptococcus mitis/oralis* | Excellent (98) | 5.02 | - | - | - | - | - | - | R | - | R |
| | **R11M** | *S. mitis* | ND | - | - | *Streptococcus mitis/oralis* | Excellent (99) | 4.82 | - | - | - | - | - | - | R | - | R |
| | **R12M** | *S. mitis* | ND | - | - | *Streptococcus mitis/oralis* | Very good (95) | 5.78 | - | - | - | - | - | - | R | - | R |
| | **R15M** | *S. parasanguinis/ cristatus* | ND | - | - | Low discrimination (repeated test: *S. thoraltensis*) | Low discrimination (50) (repeated test: 95) | 7.93 (repeated test: 2.58) | - | - | - | - | - | - | R | - | R |
| | **R16M** | *S. mitis* | ND | - | - | *Streptococcus mitis/oralis* | Excellent (99) | 5.05 | - | - | - | - | - | - | R | - | R |
| | **R18M** | *S. mitis* | ND | - | - | *Streptococcus mitis/oralis* | Excellent (99) | 5.03 | - | - | - | - | - | - | R | - | R |

+, positive; -, negative; ~, almost transparent (ambiguous result); ND, not done; ?, not readable/unclear result; R, resistant; S, susceptible

**Table S3.** Results of optochin minimum inhibitory concentration (MIC) for the development set ($n$=13) and the test set ($n$=24) isolates.

| | # | Species | MIC (µg/ml) |
|---|---|---|---|
| **Development set** | 2P | *S. pneumoniae* | 0.5 |
| | 5P | *S. pneumoniae* | 0.25 |
| | 6P | *S. pneumoniae* | 0.25 |
| | 8P | *S. pneumoniae* | 0.25 |
| | 1M | *S. mitis* | 32 |
| | 3M | *S. mitis* | 32 |
| | 4M | *S. oralis* | 64 |
| | 5M | *S. oralis* | 128 |
| | DSM20566 | *S. pneumoniae* | 0.5 |
| | DSM12643 | *S. mitis* | 8 |
| | DSM18670 | *S. pseudopneumoniae* | 0.5 |
| | DSM20627 | *S. oralis* | 32 |
| | ATCC49619 | *S. pneumoniae* | 0.5 |
| **Test set** | R2P | *S. pneumoniae* | 1 |
| | R3P | *S. pneumoniae* | 0.25 |
| | R5P | *S. pneumoniae* | 1 |
| | R6P | *S. pneumoniae* | 1 |
| | R8P | *S. pneumoniae* | 0.5 |
| | R10P | *S. pneumoniae* | 1 |
| | R11P | *S. pneumoniae* | 0.25 |
| | R13P | *S. pneumoniae* | 0.5 |
| | R14P | *S. pneumoniae* | 0.5 |
| | R15P | *S. pneumoniae* | 4 |
| | R16P | *S. pneumoniae* | 0.25 |
| | R17P | *S. pneumoniae* | 0.25 |
| | R1P | *S. pseudopneumoniae* | 1 |
| | R12P | *S. pseudopneumoniae* | 0.5 |
| | R2M | *S. mitis* | 64 |
| | R5M | *S. mitis* | 32 |
| | R6M | *S. mitis* | 64 |
| | R7M | *S. mitis* | 256 |
| | R9M | *S. oralis* | 256 |
| | R11M | *S. mitis* | 32 |
| | R12M | *S. mitis* | 64 |
| | R15M | *S. parasanguinis/ cristatus* | 128 |
| | R16M | *S. mitis* | 32 |
| | R18M | *S. mitis* | 4 |
